# Saturated fatty acid attenuates anti-obesity effect of green tea

**DOI:** 10.1038/s41598-018-28338-5

**Published:** 2018-07-03

**Authors:** Shuya Yamashita, Asami Hirashima, I-Chian Lin, Jaehoon Bae, Kanami Nakahara, Motoki Murata, Shuhei Yamada, Motofumi Kumazoe, Ren Yoshitomi, Mai Kadomatsu, Yuka Sato, Ayaka Nezu, Ai Hikida, Konatsu Fujino, Kyosuke Murata, Mari Maeda-Yamamoto, Hirofumi Tachibana

**Affiliations:** 1 Division of Applied Biological Chemistry, Department of Bioscience and Biotechnology, Faculty of Agriculture, Kyushu University, Fukuoka, 812-8581 Japan; 2 Institute of Fruit Tree and Tea Science, National Agriculture and Food Research Organization (NARO), Makurazaki, 898-0087 Japan; 3 Agri-Food Business Innovation Center, National Agriculture and Food Research Organization (NARO), Tsukuba, 305-8517 Japan

## Abstract

Green tea and its major polyphenol epigallocatechin-3-*O*-gallate (EGCG) have suppressive effect on dietary obesity. However, it remains unsolved what type of diet on which they exhibit high or low anti-obesity effect. In the present study, we investigated whether anti-obesity effect of green tea differs depending on composition of fats or fatty acids that consist high-fat (HF) diet in mouse model. Green tea extract (GTE) intake dramatically suppressed weight gain and fat accumulation induced by olive oil-based HF diet, whereas the effects on those induced by beef tallow-based HF diet were weak. GTE also effectively suppressed obesity induced by unsaturated fatty acid-enriched HF diet with the stronger effect compared with that induced by saturated fatty acid-enriched HF diet. These differences would be associated with the increasing action of GTE on expression of PPARδ signaling pathway-related genes in the white adipose tissue. Expressions of genes relating to EGCG signaling pathway that is critical for exhibition of physiological effects of EGCG were also associated with the different effects of GTE. Here, we show that anti-obesity effect of GTE differs depending on types of fats or fatty acids that consist HF diet and could be attenuated by saturated fatty acid.

## Introduction

Obesity, which is a metabolic disturbance resulting from an imbalance between fat synthesis (lipogenesis) and fat breakdown (oxidation) is prevailing worldwide at an alarming rate. It is associated with an increased risk of various health problems, such as type 2 diabetes, cardiovascular disease, and coronary heart disease^1–3^.

Tea, which is an infusion made from shoots or leaves of *Camellia sinensis* (L.) Kuntze, is one of the most popular beverages worldwide. It has been reported that daily consumption of tea is associated with decreased risks of type 2 diabetes and cardiovascular disease^4–6^. Among the major types of tea, which include unfermented tea (green tea), semi-fermented tea (oolong tea), and fermented tea (black tea), the most significant effects on human health have been observed with the consumption of green tea^7–9^. The beneficial properties of green tea are attributed to the abundance of polyphenolic compounds, i.e., catechins, including (−)-epicatechin (EC), (−)-epigallocatechin (EGC), (−)-epicatechin-3-*O*-gallate (ECG), and (−)-epigallocatechin-3-*O*-gallate (EGCG). Of these, EGCG is the most dominant in tea leaves^10^ and its numerous physiological activities, including anti-obesity, have been reported^11–13^. Suzuki *et al*. demonstrated that ‘Benifuki’, the cultivar that uniquely contains methylated-EGCG, could suppress high-fat (HF) and high-sucrose diet-induced metabolic disorder more effectively than ‘Yabukita’, the most distributed cultivar in Japan that has no methylated-EGCG^14^.

EGCG sensing system in the body has been largely revealed during these 15 years. EGCG is specifically sensed by a cell through binding to its cell surface receptor, 67-kDa laminin receptor (67LR)^15^. 67LR contributes to onset of several physiological effects of EGCG, such as anti-cancer, anti-inflammatory, adipocyte function-modifying, and vascular endothelial cell function-modifying effects^16–19^. In multiple myeloma cells, protein kinase B (Akt) and endothelial nitric oxide synthase (eNOS) are activated by EGCG, depending on 67LR, followed by induction of nitric oxide (NO) production. Subsequently, cyclic guanosine monophosphate (cGMP) is produced by NO-activated soluble guanylatecyclase (sGC), and then, acid sphingomyelinase (ASM) is activated^18^. In macrophages, EGCG inhibits lipopolysaccharide signaling via reduction of E74-like ETS transcription factor 1 expression and increase of Toll interacting protein expression, following cGMP production that depends on 67LR^19^.

Although anti-obesity effect of green tea has been reported by several groups, it remains unclear what type of diet on which green tea exhibits high or low anti-obesity effect. In the present study, we investigated whether anti-obesity effect of green tea differs depending on composition of fats or fatty acids that consist high-fat (HF) diet in mouse model. We examined the effect of green tea extract (GTE) on obesity induced by the HF diet based on olive oil that contains abundant unsaturated fatty acid (UFA), especially oleic acid, and by the HF diet based on beef tallow containing high saturated fatty acid (SFA). We also investigated the effect of GTE on obesity induced by HF diet consisted of the different ratio of UFA and SFA.

## Results

### Effect of GTE on weight gain and fat accumulation in OO-based or BT-based HF diet-fed mice

**Figure 1:**
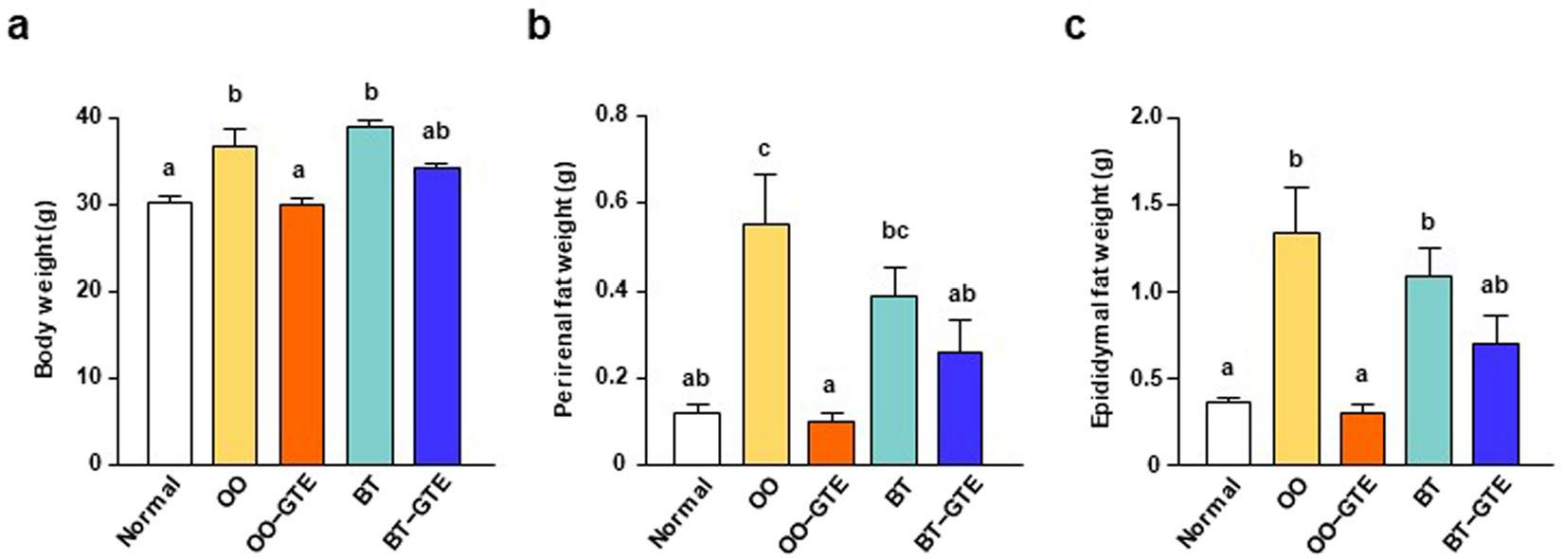
Effect of GTE on body and fat weight of OO-based or BT-based HF diet-fed mice. The body weight (**a**) and the weights of perirenal fat (**b**) and epididymal fat (**c**) of mice were measured after they were fed OO-based or BT-based HF diet (55% kcal as fat) with or without 1.0% GTE powder for 8 weeks. Values are means ± SEM, *n = *6. Different letters indicate statistically significant differences (*p* < 0.05), as indicated by a Tukey’s test.

First, we investigated the effect of GTE intake on obesity induced by two types of HF diet: olive oil (OO)-based HF diet or beef tallow (BT)-based HF diet. After acclimation for a week, mice were divided to five groups and fed the following tested diet for 8 weeks: AIN-93G diet (Normal group); OO-based HF diet (OO group); OO-based HF diet with 1.0% GTE (OO-GTE group); BT-based HF diet (BT group); BT-based HF diet with 1.0% GTE (BT-GTE group). The compositions of GTE powder and these diets were shown in Tables 1 and 2, respectively. After 8-week feeding, body weight and perirenal fat and epididymal fat weight were assessed. The energy intake of the four HF diet-fed groups (OO, OO-GTE, BT, and BT-GTE) was approximately equal, suggesting that GTE had little effect on intake of HF diet (Supplementary Fig. 1). Mice in OO and BT groups gained body weight more readily than those in Normal group, and the mean body weight of OO-GTE group was significantly lower than that of OO group, whereas that of BT-GTE group was slightly but nonsignificantly lower than that of BT group (Fig. 1a). In perirenal and epididymal fat weight, there was no significant difference between BT and BT-GTE group. OO-GTE group had lower weights of perirenal fat and epididymal fat than OO group that had higher weights than Normal group (Fig. 1b,c). These results suggest that the anti-obesity effect of GTE depends on the types of fat that consists HF diet and GTE could more effectively suppress the obesity induced by OO than that that induced by BT.

**Table 1:** Composition of GTE powder.

GTE powder (mg/g)	
(−)-Epicatechin (EC)	28.6
(−)-Epicatechin-3-*O*-gallate (ECG)	29.8
(−)-Epigallocatechin (EGC)	81.5
(−)-Epigallocatechin-3-*O*-gallate (EGCG)	111.8
(−)-Epigallocatechin-3-*O*-(3-*O*-methyl) gallate (EGCG3″Me)	21.0
(−)-Catechin (C)	6.7
(−)-Catechin gallate (CG)	2.8
(−)-Gallocatechin (GC)	22.1
(−)-Gallocatechin-3-*O*-gallate (GCG)	17.7
(−)-Gallocatechin-3-*O*-(3-*O*-methyl) gallate (GCG3″Me)	3.8
Total catechins	325.9
Caffeine	67.9

**Table 2:** Composition of the diets in Experiment 1.

	Normal	OO	OO−GTE	BT	BT−GTE
Macronutrient composition					
Protein, % of energy	19.0	17.6	17.6	17.7	17.7
Fat, % of energy	18.7	55.5	55.5	55.4	55.4
Energy, MJ/kg	15.8	20.5	20.5	20.5	20.5
Ingredient (g/kg)					
Vitamin mix^1^	10	10	10	10	10
Mineral mix^2^	35	35	35	35	35
Choline bitartrate	2.5	2.5	2.5	2.5	2.5
L-Cystin	3	3.75	3.75	3.75	3.75
Soybean oil	70	20	20	20	20
Corn oil	0	30	30	30	30
Tertiary butylhydroquinone	0.01	0.06	0.06	0.06	0.06
Sucrose	100	100	100	100	100
Casein	200	250	250	250	250
Corn starch	397.5	100	100	100	100
Pegelatinized corn starch	132	148.7	148.7	148.7	148.7
Cellulose	50	50	40	50	40
Beef tallow	0	0	0	250	250
Olive oil	0	250	250	0	0
Green tea extract powder	0	0	10	0	10

^1^Containing the following: (g/kg vitamin mix) all-*trans*-retinol acetate, 0.80; cholecalciferol, 0.25; all-rac-α-tocopherol acetate, 15; D-Biotin, 0.20; cyanocobalamin (0.1%), 2.5; folic acid, 0.20; Ca-panthothenate, 1.6; niacin, 3.0; pyridoxine-HCI, 0.70; thiamin-HCl, 0.60; riboflavin, 0.60; phylloquinone, 7.5 and sucrose, 974.66.

^2^Containing the following (g/kg mineral mix): magnesium oxide, 24; calcium carbonate, 357; potassium phosphate monobasic, 250; tripotassium citrate monohydrate, 28; sodium chloride, 74; potassium sulfate, 46.6; iron citrate, 6.06; zinc carbonate, 1.65; manganese carbonate, 0.63; copper carbonate basic, 0.324; potassium iodate, 0.01; sodium selenite, 0.01; ammonium molybdate 4H_2_O, 0.008; sodium silicate 9H_2_O, 1.45; chromium potassium sulfate 12H_2_O, 0.275; lithium chloride, 0.0174; boric acid, 0.0815; sodium fluoride, 0.0635; nickel carbonate basic 4H_2_O, 0.0306; ammonium metavanadate, 0.0066; and sucrose, 209.78.

### Effect of GTE on plasma markers in OO-based or BT-based HF diet-fed mice

We also investigated the effects of the diets on plasma markers, including triacylglycerol (TG), low-density lipoprotein cholesterol (LDL-chol), very low-density lipoprotein cholesterol (VLDL-chol), high-density cholesterol (HDL-chol), aspartate aminotransferase (AST), and alanine transaminase (ALT). Although there were no significant differences in plasma TG levels among all the groups, those in OO-GTE group tended to be lower than those in OO group and those in BT-GTE group also had a tendency to be lower than those in BT group (Table 3). There were no significant differences in the levels of LDL- and VLDL-chol among the five groups. There is a significant difference only between the BT group and the BT-GTE group in the HDL-chol levels. The plasma activities of both AST and ALT were significantly higher in OO group and BT group than in Normal group, lower in OO-GTE group than in OO group, and lower in BT-GTE group than in BT group.

**Table 3:** Levels of plasma components of mice in Experimen.

	Normal	OO	OO−GTE	BT	BT−GTE
TG (mg/dL)	68.80 ± 5.85^a^	76.20 ± 9.44^a^	67.63 ± 5.40^a^	78.54 ± 5.53^a^	72.69 ± 4.12^a^
LDL-VLDL cholesterol (μg/μL)	0.11 ± 0.01^a^	0.10 ± 0.01^a^	0.11 ± 0.01^a^	0.09 ± 0.01^a^	0.11 ± 0.01^a^
HDL cholesterol (μg/μL)	0.53 ± 0.01^ab^	0.59 ± 0.08^ab^	0.60 ± 0.04^ab^	0.50 ± 0.04^a^	0.73 ± 0.02^b^
AST (U/L)	3.07 ± 1.20^a^	10.24 ± 2.54^b^	3.27 ± 0.28^a^	6.29 ± 0.89^ab^	3.07 ± 0.16^a^
ALT (U/L)	39.15 ± 10.47^a^	109.0 ± 13.77^b^	28.73 ± 2.17^a^	99.82 ± 13.13^b^	23.87 ± 0.85^a^

Values are the means ± SEM (*n* = 6). Different letters (a and b) indicate significant difference (*p* < 0.05) by Tukey’s test.

### Effect of GTE on PPARδ-related gene expression in OO-based or BT-based HF diet-fed mice

Peroxisome proliferator-activated receptors (PPARs) are ligand-activated transcription factors in the nuclear receptor superfamily and are critical to fat metabolism^20,21^. PPARδ plays a critical role in a coordinated metabolic program by upregulating fatty acid oxidation and energy expenditure in adipocytes and myotubes^22^. PPARs first bind a specific element in the promoter region of target genes as a heterodimer with the receptor for 9-*cis* retinoic acid, RXR (retinoid X receptor). Then, they activate transcription in response to binding of the ligand. UFA, such as oleic acid, linoleic acid, and linolenic acid have been reported to have PPARδ ligand activity^23^.

**Figure 2:**
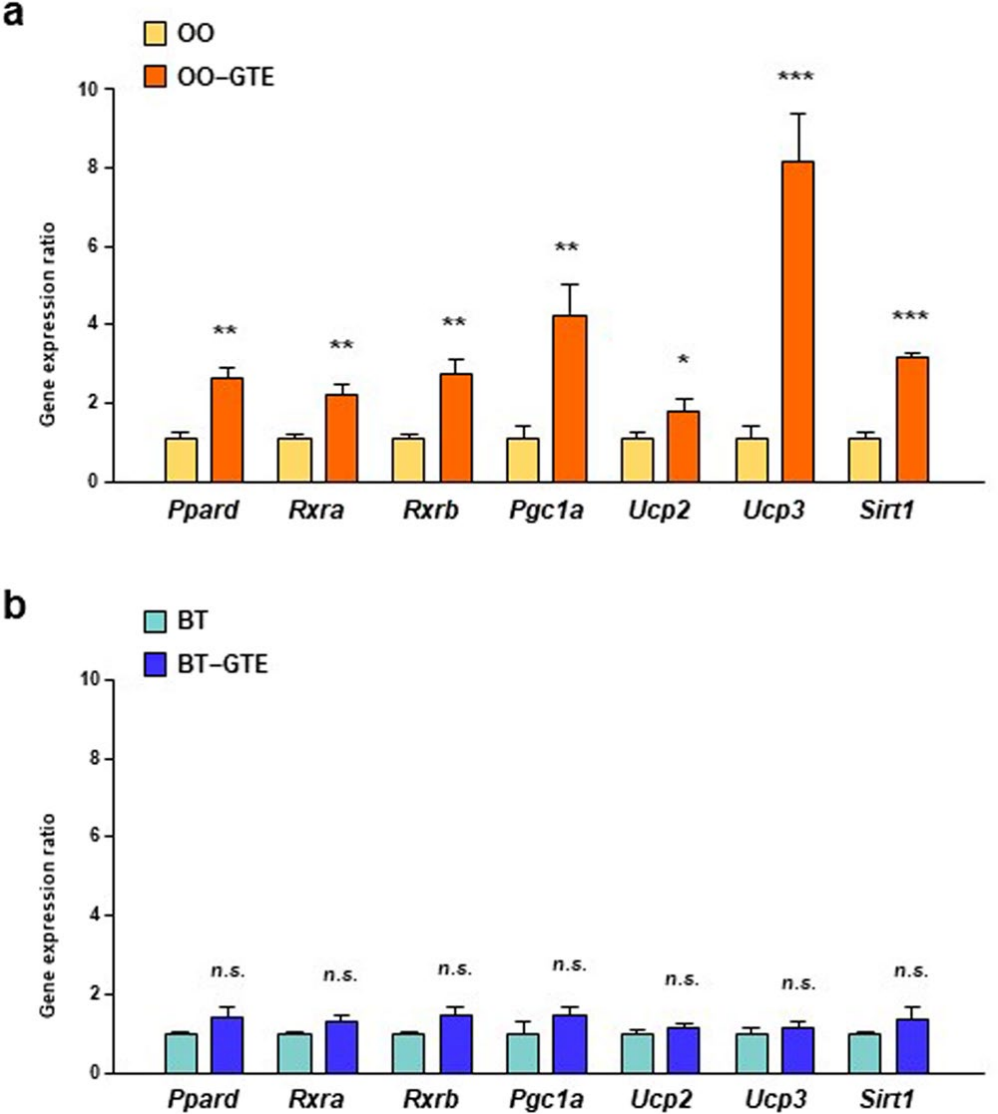
Effect of GTE on expression levels of PPARδ signaling-related genes in WAT of OO-based or BT-based HF diet-fed mice. The mRNA expression levels of *Ppard*, *Rxra*, *Rxrb*, *Pgc1a*, *Ucp2*, *Ucp3* and *Sirt1* in the perirenal adipose tissue of mice fed OO-based (**a**) or BT-based (**b**) HF diet with or without 1.0% GTE for 8 weeks were assessed using real-time quantitative PCR. Values are means ± SEM, *n = *6. *p < 0.05, **p < 0.01, ***p < 0.001, *n*.*s*. = nonsignificant by unpaired *t*-test.

For elucidation of molecular mechanism, we measured the expression levels of genes involved in PPARδ-related lipid metabolism and fatty acid oxidation, including *Ppard*, *Rxra*, *Rxrb*, *Pgc1a*, *Ucp2*, *Ucp3*, and *Sirt1* in white adipose tissue (WAT). The gene expression level of all these genes in OO-GTE group were significantly higher than those in OO group (Fig. 2a). On the other hands, there were no significant difference in these gene expression levels between BT and BT-GTE group (Fig. 2b).

**Figure 3:**
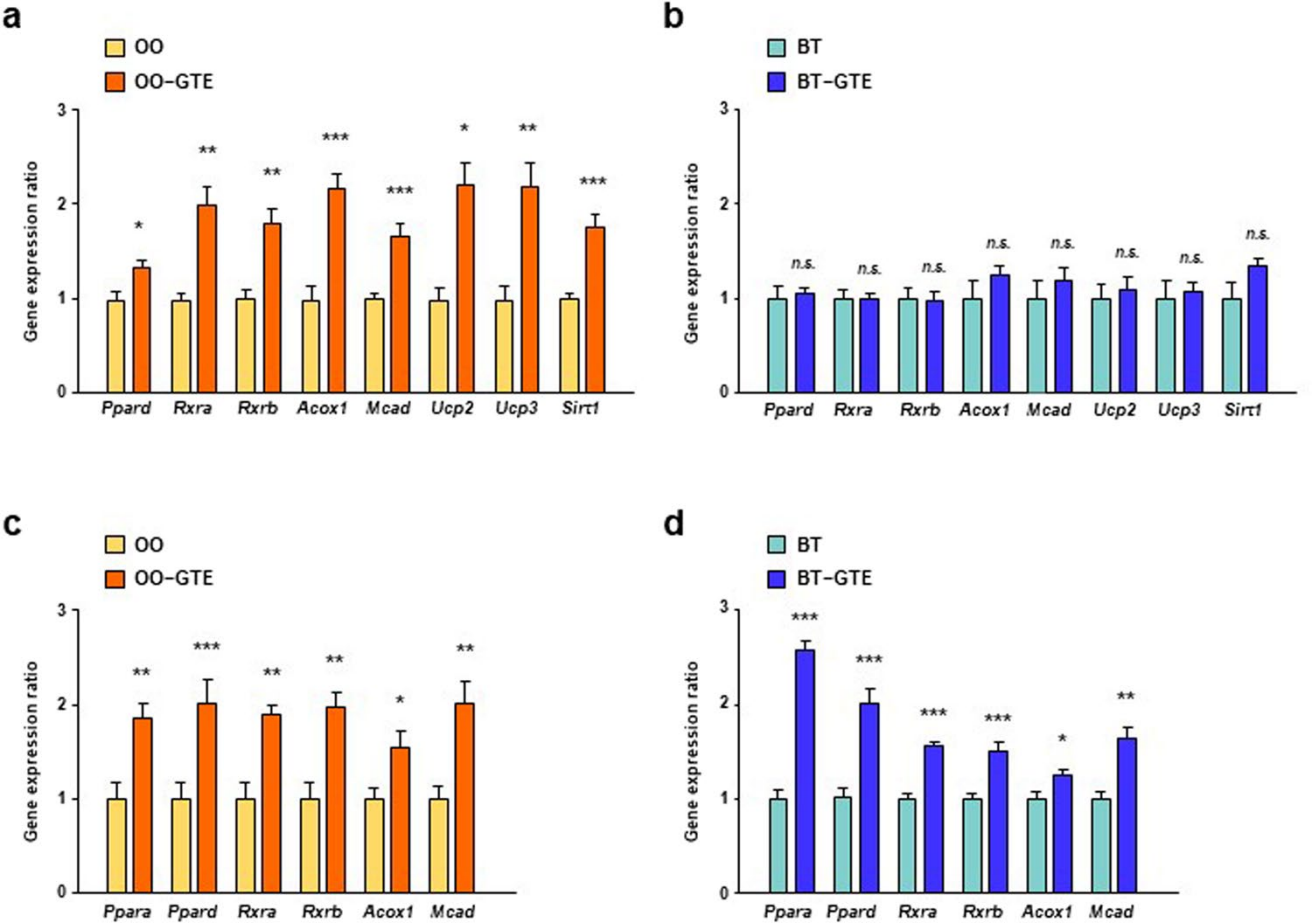
Effect of GTE on the expression levels of PPAR signaling-related genes in skeletal muscle and liver of OO-based or BT-based HF diet-fed mice. The mRNA expression levels of *Ppard*, *Rxra*, *Rxrb*, *Acox1*, *Mcad*, *Ucp2*, *Ucp3* and *Sirt1* in the skeletal muscle and of mice fed OO-based (**a**) or BT-based (**b**) HF diet with or without 1.0% GTE for 8 weeks were assessed using real-time quantitative PCR. The mRNA expression levels of *Ppara*, *Ppard*, *Rxra*, *Rxrb*, *Acox1* and *Mcad* in the liver of mice fed OO-based (**c**) or BT-based (**d**) HF diet with or without 1.0% GTE for 8 weeks were assessed by the same method. Values are means ± SEM, *n = *6. *p < 0.05, **p < 0.01, ***p < 0.001, *n*.*s*. = nonsignificant by unpaired *t*-test.

Then, the expression levels of the PPARδ-related genes, including *Ppard*, *Rxra*, *Rxrb*, *Acox1*, *Mcad*, *Ucp2*, *Ucp3*, and *Sirt1*, in skeletal muscle were measured because the tissue constitutes the largest mass in the body and is an important site of energy expenditure^24^. The expression levels of all the genes tested in OO-GTE group were significantly higher than those in OO group (Fig. 3a). On the other hands, there were no significant difference between any gene expression levels of BT group and those of BT-GTE group (Fig. 3b). We also measured the expression levels of genes, including *Ppara*, *Ppard*, *Rxra*, *Rxrb*, *Acox1*, and *Mcad*, in liver. The expression levels of all these genes were higher in OO-GTE group than in OO group and were also higher in BT-GTE group than in BT group (Fig. 3c,d).

### Effect of GTE on weight gain and fat accumulation in UFA-enriched or SFA-enriched HF diet-fed mice

Edible fat and oil contain lots of chemical constituents other than fatty acids. BT contains choline and vitamin D and OO contains vitamin E, carotenoids, and phenolic compounds^25^. So, we next investigated whether simple difference between UFAs and SFAs can affect anti-obesity effect of GTE by preparing the two types of HF diet: one was constituted with a large quantity of UFAs (UFA-enriched HF diet: SFAs 2.7% of energy; UFAs 52.4% of energy) and the other was composed with a large amount of SFAs (SFA-enriched HF diet: SFAs 30% of energy; UFAs 25% of energy). Only oleic acid was replaced to β-corn starch in UFA-enriched HF diet. For SFA-enriched HF diet, palmitic acid and stearic acid were replaced to a part amount of β-corn starch. The mice were divided five groups and fed the following diets for 8 weeks: AIN-93G diet (Normal group); UFA-enriched HF diet (UFA group); UFA-enriched HF diet supplemented with 1.0% GTE (UFA-GTE); SFA-enriched HF diet (SFA group); SFA-enriched HF diet supplemented with 1.0% GTE (SFA-GTE group). The component composition of GTE powder used in Experiment 2 was approximately same as the one used in Experiment 1 (Supplementary Table S1). The composition of the experimental diets was shown in Table 4.

**Table 4:** Composition of the diets in Experiment 2.

	Normal	UFA	UFA−GTE	SFA	SFA−GTE
Macronutrient composition					
Protein, % of energy	19.6	14.5	14.5	14.5	14.5
Fat, % of energy	17.8	55.4	55.4	55.4	55.4
Saturated fatty acid, % of energy	2.7	2.7	2.7	30.0	30.0
Unsaturated fatty acid, % of energy	14.2	52.4	52.4	25.0	25.0
Energy, MJ/kg	15.4	20.1	20.1	20.1	20.1
Ingredient (g/kg)					
Vitamin mix^1^	10	10	10	10	10
Mineral mix^2^	35	35	35	35	35
Choline bitartrate	2.5	2.5	2.5	2.5	2.5
L-Cystin	3	3	3	3	3
Soybean oil	70	94	94	94.5	94.5
Tertiary butylhydroquinone	0.01	0.06	0.06	0.06	0.06
Sucrose	100	100	100	100	100
Casein	200	200	200	200	200
Corn starch	397.5	160.4	160.4	160.9	160.9
Pegelatinized corn starch	132	132	132	132	132
Cellulose	50	50	40	50	40
Palmitic acid	0	0	0	100	100
Stearic acid	0	0	0	52	52
Oleic acid	0	213	213	60	60
Green tea extract powder	0	0	10	0	10

^1^Containing the following: (g/kg vitamin mix) all-*trans*-retinol acetate, 0.80; cholecalciferol, 0.25; all-rac-α-tocopherol acetate, 15; D-Biotin, 0.20; cyanocobalamin (0.1%), 2.5; folic acid, 0.20; Ca-panthothenate, 1.6; niacin, 3.0; pyridoxine-HCI, 0.70; thiamin-HCl, 0.60; riboflavin, 0.60; phylloquinone, 7.5 and sucrose, 974.66.

^2^Containing the following (g/kg mineral mix): magnesium oxide, 24; calcium carbonate, 357; potassium phosphate monobasic, 250; tripotassium citrate monohydrate, 28; sodium chloride, 74; potassium sulfate, 46.6; iron citrate, 6.06; zinc carbonate, 1.65; manganese carbonate, 0.63; copper carbonate basic, 0.324; potassium iodate, 0.01; sodium selenite, 0.01; ammonium molybdate 4H_2_O, 0.008; sodium silicate 9H_2_O, 1.45; chromium potassium sulfate 12H_2_O, 0.275; lithium chloride, 0.0174; boric acid, 0.0815; sodium fluoride, 0.0635; nickel carbonate basic 4H_2_O, 0.0306; ammonium metavanadate, 0.0066; and sucrose, 209.78.

**Figure 4:**
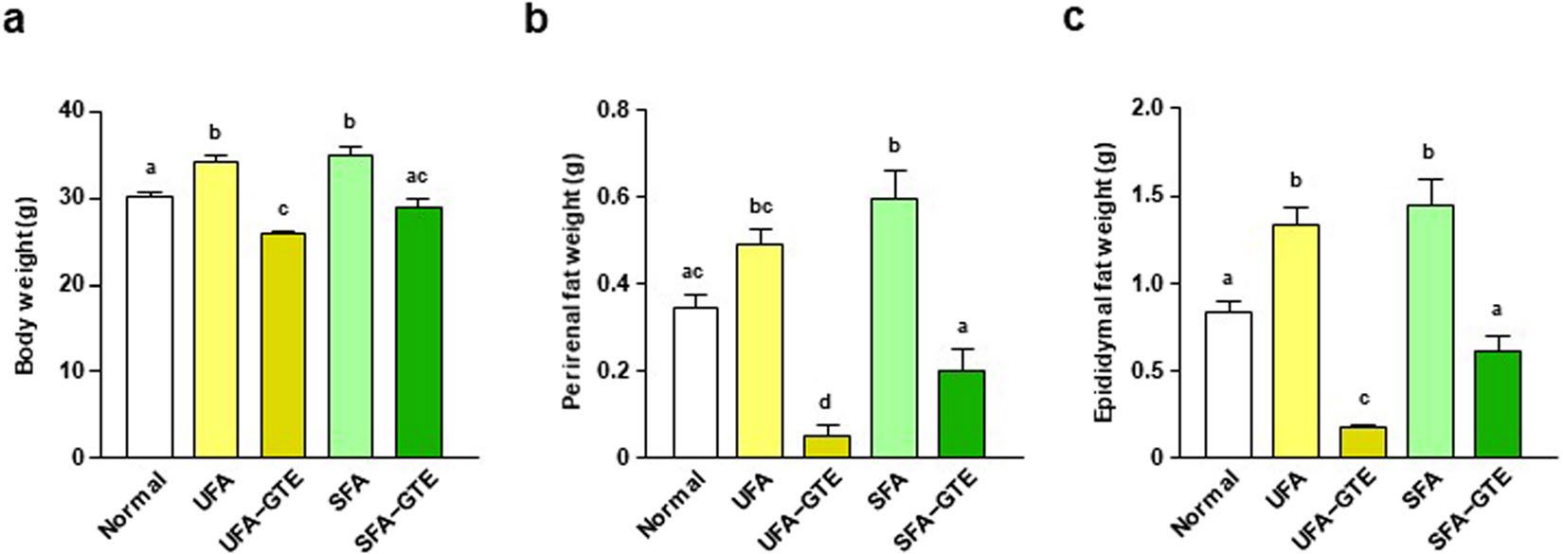
Effect of GTE on body and fat weight of UFA-enriched or SFA-enriched HF diet-fed mice. The body weight (**a**) and the weights of perirenal fat (**b**) and epididymal fat (**c**) of mice were measured after they were fed UFA-enriched or SFA-enriched HF diet (55% kcal as fat) with or without 1.0% GTE powder for 8 weeks. Values are means ± SEM, *n = *7. Different letters indicate statistically significant differences (*p* < 0.05), as indicated by a Tukey’s test.

The body weight of UFA and SFA group after 8-week feeding were approximately equal and both were higher than that of Normal group. The body weight of UFA-GTE group and of SFA-GTE group were significantly lower than that of UFA group and of SFA group, respectively. The body weight of UFA-GTE group had tended to be low compared with that of SFA-GTE group (*P* = 0.0526) (Fig. 4a). Perirenal fat weight of UFA-GTE group and of SFA-GTE group were lower than that of UFA group and of SFA group, respectively. Perirenal fat weight of UFA-GTE group was lower than that of SFA-GTE group (Fig. 4b). The result of the epididymal fat weight was similar to that of perirenal fat (Fig. 4c).

### Effect of GTE on PPARδ-related gene expression in UFA-enriched or SFA-enriched HF diet-fed mice

**Figure 5:**
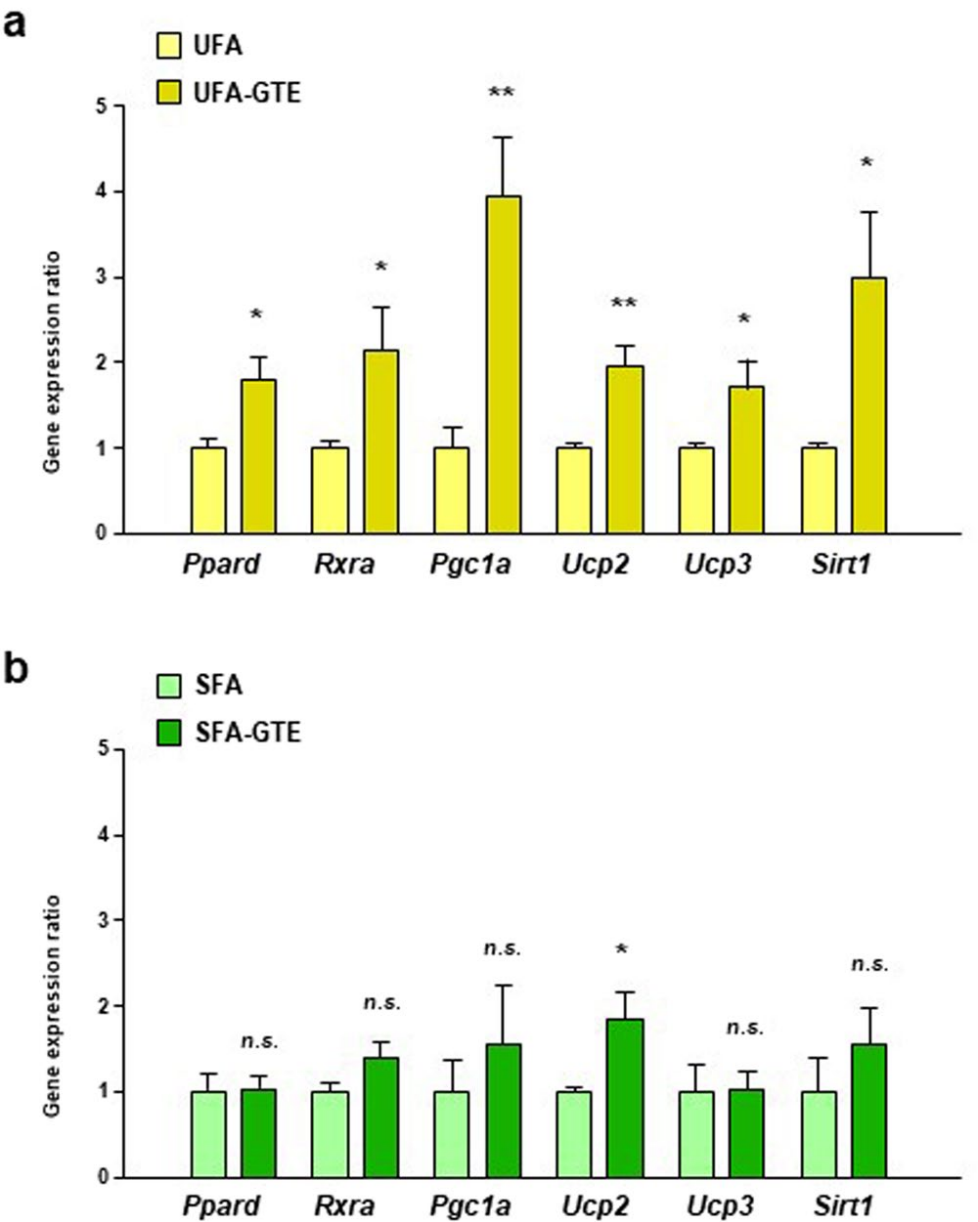
Effect of GTE on expression levels of PPARδ signaling-related genes in WAT of UFA-enriched or SFA-enriched HF diet-fed mice. The mRNA expression levels of *Ppard*, *Rxra*, *Pgc1a*, *Ucp2*, *Ucp3*, and *Sirt1* in the perirenal adipose tissue of mice fed UFA-enriched (**a**) or SFA-enriched (**b**) HF diet with or without 1.0% GTE for 8 weeks were assessed using real-time quantitative PCR. Values are means ± SEM, *n = *5–6. *p < 0.05, **p < 0.01, ***p < 0.001, *n*.*s*. = nonsignificant by unpaired *t*-test.

The expression levels of genes involved in PPARδ signaling pathway in WAT were also measured in the second *in-vivo* experiment. The expression levels of *Ppard*, *Rxra*, *Pgc1a*, *Ucp2*, *Ucp3* and *Sirt1* in WAT of UFA-GTE group were significantly higher than those of UFA group (Fig. 5a). On the other hands, only *Ucp2* expression was higher in SFA-GTE group than those of SFA group (Fig. 5b).

### Effect of GTE on EGCG signaling-related gene expression in mice fed four experimental diet

**Figure 6:**
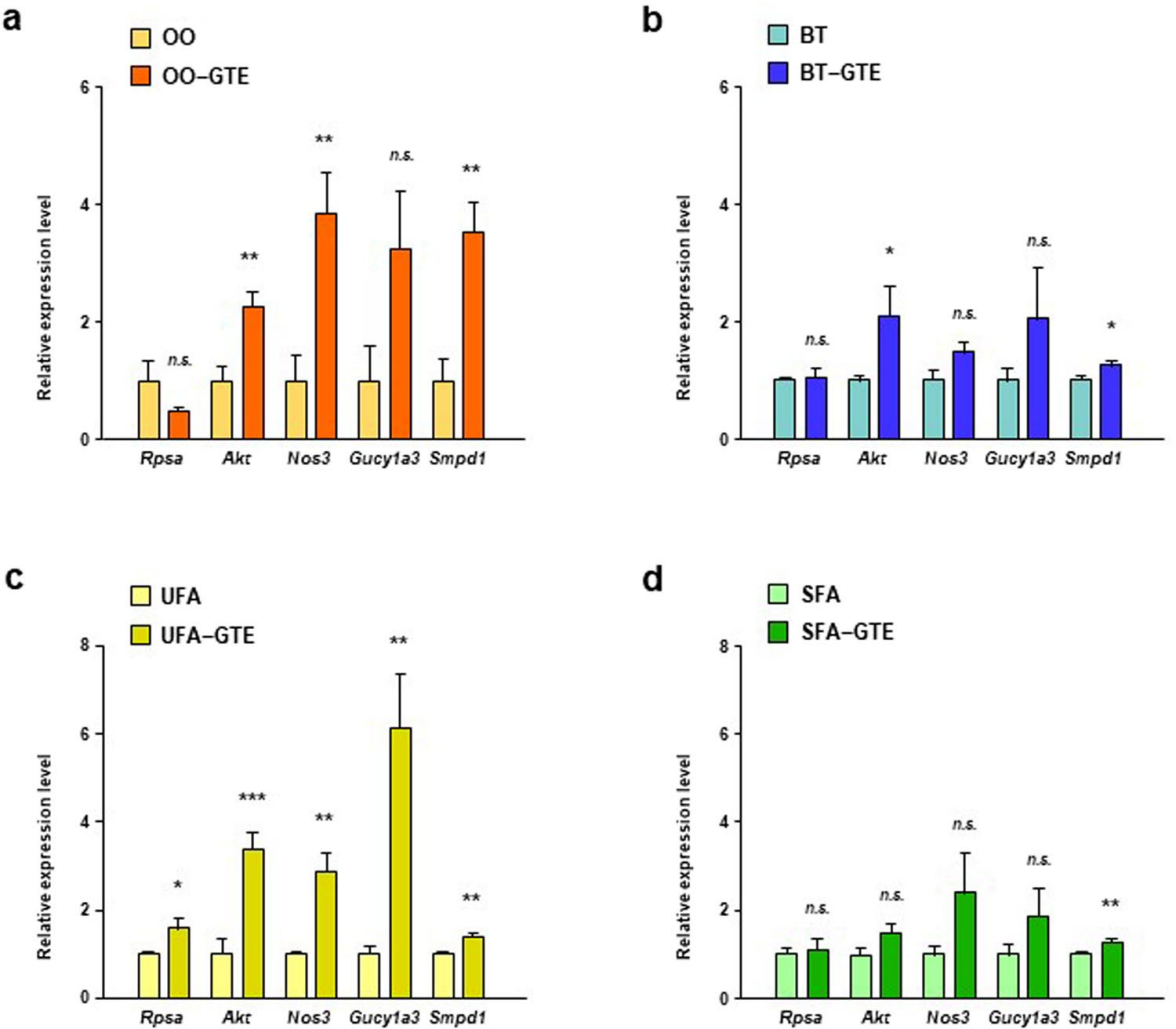
Effect of GTE on expression levels of EGCG signaling-related genes in WAT of mice fed OO-based, BT-based, UFA-enriched or SFA-enriched HF diet. The mRNA expression levels of *Rpsa*, *Akt*, *Nos3*, *Gucy1a3* and *Smpd1* in the perirenal adipose tissue of mice fed OO-based (**a**), BT-based (**b**), UFA-enriched (**c**) or SFA-enriched (**d**) HF diet with or without 1.0% GTE for 8 weeks were assessed using real-time quantitative PCR. Values are means ± SEM, *n = *6. *p < 0.05, **p < 0.01, ***p < 0.001, *n*.*s*. = nonsignificant by unpaired *t*-test.

EGCG sensing system in the body is a key for the onset of physiological effects of EGCG. 67LR is a cell surface receptor for EGCG and mediates various functionalities of EGCG^16–19^. After EGCG binds to 67LR, Akt and eNOS activates in turns, followed by production of NO. NO activated-sGC produces cGMP, and then ASM gets active^18^. Silencing of EGCG signaling-related genes canceled the EGCG actions, indicating the significance of their expression levels for EGCG effects^17–19^. We investigated the effect of GTE on the expression level of EGCG signaling-related genes in WAT of mice in both of two *in-vivo* experiments for elucidating the mechanism of increasing effect of GTE on PPARδ-related gene expression in OO-based and UFA-enriched HF diet. The expression levels of *Akt*, *Nos3*, and *Smpd1* of OO-GTE group were significantly higher in those of OO group (Fig. 6a). Only *Akt* and *Smpd1* expression level of BT-GTE group were significantly higher than that of BT group but the difference was very small (Fig. 6b). The expression levels of all the tested genes of UFA-GTE group were higher than those of UFA group (Fig. 6c). On the other hands, only *Smpd1* expression level of SFA-GTE group were slightly higher than that of SFA group (Fig. 6d).

## Discussion

In the present study, we investigated the effect of GTE intake on obesity induced by four types of HF diets that were mainly consisted with different types of fat, OO or BT, or with different composition of fatty acids, UFA or SFA. A lot of researches revealed anti-obesity effects of green tea and/or catechins on animal models by investigating those induced by HF diet mainly consisted of single animal fat, such as beef tallow or lard^13,14,26^. This is the first report investigating the difference of fat types in the anti-obesity action of green tea. In the present study, GTE significantly suppressed body weight gain and fat accumulation induced by OO-based HF diet whereas the suppressive effect on those induced by BT-based HF diet was weak. Although obesity would have developed by combination of several ingredients contained in the experimental diet, such as OO or BT, soybean oil, corn oil, sucrose, casein, corn starch, and so on, the different efficacy of GTE on these two different diets would be due to the difference of OO and BT because other components that were contained in these diets were common.

GTE increased the gene expression of *Ppard*, *Rxra*, *Rxrb*, *Pgc1a*, *Ucp2*, *Ucp3*, and *Sirt1* in WAT of mice fed OO-based HF diet. PPARδ has been reported to play a critical role in adipose tissue increase and whole-body lipid catabolism^22^. UFAs have been suggested to function as dietary ligands for PPARδ ligands^23^. RXR is known as a receptor for 9-*cis* retinoic acid that was reported to inhibit adipogenesis by activating RXR^27^. RXR is also able to contribute to the functions of PPARs by functioning as heterodimeric partners, as indicated by the finding that RXR antagonist reversed anti-inflammatory effects of PPARγ^28^. GTE-induced upregulation of *Ppard*, *Rxra*, and *Rxrb* expression might increase the function of the dietary PPARδ ligand, such as oleic acid contained in OO. *Ucp2* and *Ucp3* are PPARδ target gene and encode uncoupling protein 2 and uncoupling protein 3, respectively, that seem to be potential regulators of mitochondrial energy metabolism^29^. The greater expression of WAT *Ucp2* and *Ucp3* in the OO-GTE group than in the OO group suggests that GTE promotes energy expenditure in WAT. PGC1α forms a complex with PPARs and controls the expression of genes that encode enzymes associated with both mitochondrial biogenesis and fatty acid metabolism^30,31^. Sirtuin 1, a nicotinamide adenosine dinucleotide (NAD)-dependent protein deacetylase encoded by *Sirt1*, functions as an important regulator of cellular stress response and energy metabolism. Sirtuin 1 is involved in protection against metabolic disorders and in the prevention of obesity-induced adipose tissue inflammation^32^. GTE intake could improve energy metabolism by inducing the expression of *Pgc1a* and *Sirt1* in WAT of mice fed OO-based HF diets.

GTE upregulated the expression of *Ppard*, *Rxra*, and *Rxrb* in the skeletal muscle of OO-fed mice, which indicates that GTE could promote the usability of UFAs, including oleic acid, in skeletal muscle. The activation of skeletal PPARδ modulates the expression of fat-burning genes, such as *Ucps*, and induces fatty acid beta-oxidation, thereby attenuating metabolic syndrome^33,34^. *Acox1*, which encodes acyl-CoA oxidase 1 (ACOX1), and *Mcad*, which encodes medium-chain acyl-CoA dehydrogenase (MCAD), are also PPAR target genes that are involved in fatty acid beta-oxidation^35,36^. The greater expression levels of PPARδ target genes (*Ucp2*, *Ucp3*, *Acox1*, and *Mcad*) in the skeletal muscle of OO-GTE group, when compared to those of OO group, suggests that GTE enhances the transcription activity of PPARδ and induces fat metabolism in the skeletal muscle of OO-based HF diet-fed mice. Sirtuin 1 regulates mitochondrial gene transcription and biogenesis in skeletal muscle^37,38^. Mitochondria convert nutrients into energy, and the upregulation of mitochondrial biogenesis promotes fatty acid beta-oxidation^39^. GTE-induced *Sirt1* expression might contribute to mitochondrial biogenesis and beta-oxidation in OO-based HF diet-fed mice.

In a liver, PPARs modulates the expression of genes, such as *Acox1* and *Mcad*, that are associated with fatty acid oxidation^40^. The expression levels of *Ppard* and PPAR target genes in a liver were increased by GTE intake in both mice fed OO-based HF-diet and BT-based one. The results of plasma AST and ALT levels indicate that GTE intake could protect liver from hepatotoxicity induced by OO-based HF diet and BT-based one. These results suggest that GTE would improve liver function in both OO-based diet-fed mice and BT-based diet-fed mice, but the improvement would not contribute to the difference of GTE anti-obesity effect on between OO-based HF diet and BT-based HF diet.

GTE also significantly suppressed increase of body weight and adiposity induced by UFA-enriched HF diet and the effect was stronger than those induced by SFA-enriched HF diet. These data suggest that anti-obesity action of GTE depends on the composition of fatty acids that consist HF diet and UFA-enriched diet would be more beneficial for enjoying the GTE effect than SFA-enriched diet. GTE commonly upregulated the mRNA expression level of *Ppard*, *Rxra*, *Pgc1a*, *Ucp2*, *Ucp3* and *Sirt1* in WAT in the OO-based HF diet-fed mice and the UFA-enriched HF diet-fed mice, suggesting that the mechanism for the effectiveness of GTE on these diet-induced fat accumulations might be due to promotion of energy expenditure through activation of PPARδ pathway in WAT and through ingestion of large amount of UFA, or PPAR ligands, into the body. Conversely, SFA could dilute the anti-obesity effect of GTE because the activation action of GTE on PPARδ pathway in WAT was hardly observed in SFA-enriched HF diet-fed mice. Since obesity induces chronic inflammation within adipose tissue^41^ and EGCG plays a central role in the anti-inflammatory effects of green tea polyphenols^19^, GTE also could effectively prevent inflammation induced by excessive intake of diet consisted with UFA as main fatty acid. Although the effect of oleic acid was investigated in the present study, polyunsaturated fatty acids (PUFA), such as linolenic acid, linoleic acid, arachidonic acid, or eicosapentaenoic acid, might also exhibit similar combination effect with green tea because they have been reported to have PPAR ligand activity similar to oleic acid^23^.

EGCG is the most dominant polyphenol in tea leaves and has numerous physiological activities including anti-obesity one^10–13^. EGCG signaling pathway in which 67LR functions as an entrance contributes to exhibitions of several physiological effects of EGCG^16^. In multiple myeloma cells, EGCG activates Akt and eNOS through 67LR, followed by inducing production of NO. Subsequently, cGMP is produced, depending on sGC, followed by activation of ASM^18^. 67LR, Akt, eNOS, sGC and ASM are encoded by *Rpsa*, *Akt*, *Nos3*, *Gucy1a3* and *Smpd1*, respectively. GTE intake significantly increased the expression levels of EGCG signaling pathway-related genes in WAT of mice of OO-based and UFA-enriched HF diet whereas it slightly affected those of mice fed BT-based and SFA-enriched HF diet. These results suggest that EGCG would be one of the active compounds and that acceleration of EGCG signaling in WAT would contribute to the effectiveness of anti-obesity and PPARδ pathway-activating effect of GTE in mice fed OO-based and UFA-enriched HF diet.

The ratio of SFA and UFA in the experimental diets were as follows: OO-based HF diet, SFA 7.7% calorie and UFA 46.2% calorie; BT-based HF diet, SFA 24.3% calorie and UFA 28.7% calorie; UFA-enriched HF diet, SFA 2.7% calorie and UFA 52.4% calorie; SFA-enriched HF diet, SFA 30.0% calorie and UFA: 25.0% calorie. Although GTE were able to exhibit a certain degree of suppressive effect on obesity induced by BT-based HF diet or SFA-enriched HF diet (SFA 24.3% or 30.0% calorie, respectively), drinking the required amount of GTE (equivalent to 10 cups/day for human diet of 2,000 kcal/day) in daily life is difficult. Therefore, it might be possible to enjoy anti-obesity action of drinking green tea in realistic quantity by replacing SFA-rich diet with UFA-rich diet.

OO is the most representative food of traditional Mediterranean Diet (MedDiet). Increasing evidence suggests that UFAs as a nutrient, OO as a food, and the MedDiet as a food pattern are associated with a decreased risk of cardiovascular disease, obesity, metabolic syndrome, type 2 diabetes and hypertension^42,43^. Intervention trial demonstrated that consumption of extra virgin OO contained in Western-diet reduced total body fat and blood pressure in the independent manner of caloric restriction^44^. In the present study, the combination of GTE and OO effectively suppressed diet-induced obesity, indicating that MedDiet perhaps could promote the anti-obesity effect of GTE.

In conclusion, this study demonstrates that GTE can effectively suppress obesity induced by OO-based HF diet and UFA-enriched HF diet by activation of PPARδ pathway in WAT. On the other hands, anti-obesity effect of GTE was less effective on SFA-enriched HF diet than on UFA-enriched HF diet, suggesting that SFA would attenuate the GTE effect through suppressing the PPARδ pathway activation effect of GTE. These differences of GTE effect on obesity induced by OO-based, BT-based, UFA-enriched, and SFA-enriched HF diet, would be caused by presence or absence of upregulation of genes relating to EGCG signaling pathway. Drinking green tea and replacing animal fat rich in SFA with vegetable oil rich in UFA, such as olive oil, could be a useful dietary habit to prevent obesity.

## Methods

### Animals and diets

12-week-old and 9-week-old male C57BL/6 J mice were purchased in Experiment 1 and Experiment 2, respectively, from Charles River (Kanagawa, Japan). They were maintained in a temperature- and humidity-controlled room with a 12-h-light–dark cycle (light from 8 am to 8 pm). All mice were acclimated for 1 week while being fed an AIN-93G diet. AIN-93G and HF diets were obtained from KBT Oriental (Tokyo, Japan). Mice in Experiment 1 were assigned the five groups: Normal group that was fed AIN-93G diet (18.7% of energy as fat), OO group that was fed an OO-based HF diet (55.5% of energy as fat), OO-GTE group that was fed an OO-based HF diet supplemented with 1.0% GTE, BT group that was fed a BT-based HF diet (55.4% of energy as fat), or BT-GTE group that was fed a BT-based HF diet supplemented with 1.0% GTE. In Experiment 2, mice were assigned the five groups: Normal group that was fed AIN-93G diet (2.7% of energy as SFA and 14.2% of energy as UFA); UFA group that was fed UFA-enriched HF diet (2.7% of energy as SFA and 52.1% of energy as UFA); UFA-GTE group that was fed UFA-enriched HF diet supplemented with 1.0% GTE; SFA group was fed SFA-enriched HF diet (30.0% of energy as SFA and 25.0% of energy as UFA); SFA-GTE group that was fed SFA-enriched HF diet supplemented with 1.0% GTE. ‘Benifuki’ was used as the tea cultivar and the extract was prepared from the leaves of the plant using boiling water followed by freeze-drying. The composition of GTE powder was determined by high-performance liquid chromatography (HPLC) (Table 1 and Supplementary Table S1). The dietary level of green tea at 1.0% was equivalent to human intakes of 10 cups (2.0 g of green tea/cup) per day as estimated based on an energy intake of 2,000 kcal/day. Mice were fed the diets every two days and were weighed every week. At the end of 8 weeks of feeding, mice were anesthetized under isoflurane vapor after overnight food deprivation. Blood samples were collected into tubes from ventral aorta. Plasma was collected after centrifugation (2,000 × g for 15 min at 4 °C) and stored at −80 °C. After blood collection, mice were killed by isoflurane overdose. Visceral adipose tissues (peritoneal and epididymal depots) were harvested, rinsed, and weighed. This experiment was carried out according to the guidelines for animal experiments at the Faculty of Agriculture, Kyushu University. The study protocol was approved by the Animal Care and Use Committee of Kyushu University, Fukuoka, Japan. The approval number for Experiment 1 and Experiment 2 are A22-146 and A28-159, respectively.

### Composition of green tea extract powder

The GTEs were obtained from Asahi Soft Drinks (Asahi Soft Drinks Company, Ltd., Ibaraki, Japan). The GTE was obtained from the green tea leaves using seething water (over 70 °C, ~30 min) and freeze-drying techniques. The amount of catechins and caffeine in the GTE powder were determined by high-performance liquid chromatography, as previously described^14^. Briefly, samples were immersed in 50% ethanol containing 1% H_3_PO_4_, disrupted using ultrasonic apparatus for 30 min at 20 °C, and then filtered through a membrane filter (pore size, 0.45 μm). HPLC was undertaken using a Shimadzu LC-10A pump coupled to an ultraviolet detector (SPD-M10Avp; Shimadzu, Kyoto, Japan) and a reverse-phase Wakopak Navi C18-5 column (4.6 mm i.d. × 150 mm; granule diameter, 5 μm; Wako Pure Chemical Industries, Kyoto, Japan). Elution consisted of a 2–45 min linear gradient from 0 to 80% of solvent B and solvent A. Solvent A consisted of distilled water/phosphoric acid/acetonitrile (400: 10: 1 v/v), and solvent B consisted of methanol/solvent A (1: 2 v/v). Samples were eluted at 1 mL/min at 40 °C. The detection wavelength was 272 nm. The amounts of catechins in tea extracts were measured by comparing the peak area of each catechin in the tea extract with that of a standard preparation that contained a fixed quantity of (−)-epicatechin (EC), (−)-epicatechin-3-*O*-gallate (ECG), (−)-epigallocatechin (EGC), (−)-epigallocatechin-3-*O*-gallate (EGCG), (−)-epigallocatechin-3-*O*-(3-*O*-methyl) gallate (EGCG3″Me), (−)-catechin (C), (−)-catechin gallate (CG), (−)-gallocatechin (GC), (−)-gallocatechin-3-*O*-gallate (GCG), (−)-gallocatechin-3-*O*-(3-*O*-methyl) gallate (GCG3″Me), and caffeine (Table 1 and Supplementary Table S1).

### Biochemical analyses of plasma

Plasma levels of triglyceride (TG), aspartate aminotransferase (AST), and alanine transaminase (ALT) were measured using the TG E-test (Wako Chemical, Osaka, Japan) and the transaminase CII-test (Wako Chemical), respectively. High-density lipoprotein cholesterol (HDL-chol) and low-density (LDL-chol) and very low-density lipoprotein-cholesterol (VLDL-chol) were assayed using HDL-C and LDL-C/VLDL-C Quantification Kit (BioVision, Milpitas, CA, USA).

### Real-time quantitative polymerase chain reaction (PCR)

The tissue samples were stored at −80 °C until use. The total RNA was extracted from the tissue samples using TRI Reagent (Sigma-Aldrich, St Louis, MO) following the manufacture’s instructions. The complementary DNA (cDNA) was synthesized from the total RNA (400 ng) using PrimeScript RT reagent Kit (Takara Bio, Tokyo, Japan). Gene expression was analyzed by real-time quantitative PCR using SYBR green procedure and CFX96 or CFX384 real-time PCR analysis system (BIO-RAD, Hercules, CA). The values of mRNA expressions of each gene were normalized relative to *Actb* as an internal control. The specific primer sequences were given in Supplementary Table S2.

### Statistical analyses

The results are presented as means ± standard error of the mean (SEM). Statistical analysis was performed using GraphPad Prism software by one-way analysis of variance (ANOVA), followed by Tukey’s post hoc test or unpaired *t*-test according to the analysis.

## Electronic supplementary material

Supplementary Information
