# Supplementary material for: Saturated fatty acid attenuates anti-obesity effect of green tea
Source: Sci Rep. 2018 Jul 3;8:10023. doi: 10.1038/s41598-018-28338-5 (PMC6030063; doi:10.1038/s41598-018-28338-5)
Supplement: Supplementary file 1 — Supplementary Information [file 41598_2018_28338_MOESM1_ESM.pdf]

Saturated fatty acid attenuates anti-obesity effect of green tea

Shuya Yamashita[1,2+], Asami Hirashima[1+], I-Chian Lin[1], Jaehoon Bae[1], Kanami Nakahara[1], Motoki Murata[1], Shuhei Yamada[1], Motofumi Kumazoe[1], Ren Yoshitomi[1], Mai Kadomatsu[1], Yuka Sato[1], Ayaka Nezu[1], Ai Hikida[1], Konatsu Fujino[1], Kyosuke Murata[1], Mari Maeda-Yamamoto[3] and Hirofumi Tachibana[1,*]

[1] Division of Applied Biological Chemistry, Department of Bioscience and Biotechnology, Faculty of Agriculture, Kyushu University, Fukuoka 812-8581, Japan

[2] Institute of Fruit Tree and Tea Science, National Agriculture and Food Research Organization (NARO), Makurazaki 898-0087, Japan

[3] Agri-Food Business Innovation Center, National Agriculture and Food Research Organization (NARO), Tsukuba 305-8517, Japan

* Corresponding author. E-mail: tatibana@agr.kyushu-u.ac.jp

[+] These authors equally contributed to this work.

Supplementary Table S1. Composition of GTE powder used in Experiment 2

	GTE powder (mg/g)
(−)-Epicatechin (EC)	23.3
(−)-Epicatechin-3-*O*-gallate (ECG)	32.4
(−)-Epigallocatechin (EGC)	69.9
(−)-Epigallocatechin-3-*O*-gallate (EGCG)	129.6
(−)-Epigallocatechin-3-*O*-(3-*O*-methyl) gallate (EGCG3''Me)	17.5
(−)-Catechin (C)	8.2
(−)-Catechin gallate (CG)	2.6
(−)-Gallocatechin (GC)	22.0
(−)-Gallocatechin-3-*O*-gallate (GCG)	19.8
(−)-Gallocatechin-3-*O*-(3-*O*-methyl) gallate (GCG3''Me)	3.2
Total catechins	328.5
Caffeine	78.4

Supplementary Table S2. Quantitative realtime PCR primers

Gene	Accession No.	Forward Primer	Reverse Primer
Ppard	NM_011145.3	5'-CAGCAGACAGCCCGTTGGT-3'	5'-CTTTGGATGGCTCATGTCTTTGAA-3'
Ppara	NM_001113418.1	5'-CCAATGTCCTGGCCACATTTC-3'	5'-GCCTGTCCTCAGGTGTGTCTGTA-3'
Rxra	NM_001290481.1	5'-ACCAATGGCTAGTCATCTGCTGTC-3'	5'-GTCGTCAGTGGGCAGTCTCTGTA-3'
Rxrb	NM_001205214.1	5'-ATGCGTGACATGAGGATGGA-3'	5'-GAAGACGTAACAGCAGCTTGG-3'
Ucp2	NM_011671.4	5'-GCAAGCATGTGTATGGCACAGTAA-3'	5'-AAATGTGGGCCTTCGGTCAG-3'
Ucp3	NM_204107.1	5'-CTGCACCGCCAGATGAGTTT-3'	5'-ATCATGGCTTGAAATCGGACC-3'
Sirt1	NM_019812.2	5'-GCAGACGTGGTAATGTCCAAACAG-3'	5'-ACATCTTGGCAGTATTTGTGGTGAA-3'
Acox1	NM_015729.3	5'-GCTTGGAAACCACTGCCACA-3'	5'-CTGAGCCAGGACTATCGCATGA-3'
Mcad	NM_007382.4	5'-TGATGTGGCGGCCATTAAGA-3'	5'-CGGCTTCCACAATGAATCCAG-3'
Pgc1a	NM_008904.1	5'-GCGCCGTGTGATTTACGTT-3'	5'-CGGTAGGTGATGAAACCATAGCTG-3'
Rpsa	NM_011029.3	5'-CTGTGCCCATCCAGCAGTTC-3'	5'-AGCTGCTGACCAATCCTCAGTG-3'
Akt1	NM_001165894.1	5'-CATCGTGTGGCAGGATGTGTA-3'	5'-ACCTGGTGTCAGTCTCAGAGGTG-3'
Nos3	NM_008713.	5'-ATTCTGGCAAGACAGACTACACGA-3'	5'-TCCCGGTAGAGATGGTCCAG-3'
Gucy1a3	NM_001356987.1	5'-AGTTCTAACCCAAGCGGTGTGAAG-3'	5'-GTGTACACAGGCGACACAAGTGAG-3'
Smpd1	NM_011421.2	5'-CCAGGACATTGTCTTAAGAGCTGGA-3'	5'-AGCTAGTGGTCGGCTCAGAGTTTC-3'
Actb	NM_001124235.1	5'-CATCCGTAAAGACCTCTATGCCAA-3'	5'-ATGGAGCCACCGATCCACA-3'

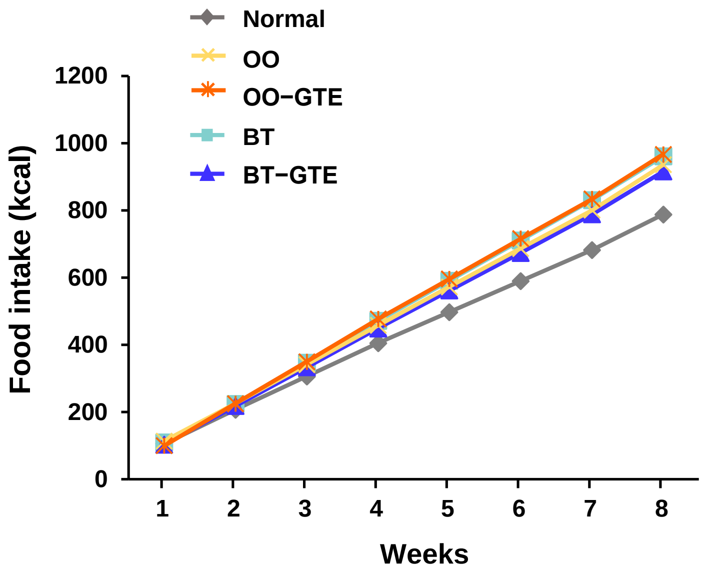

Supplementary Figure 1. Food intake of the tested groups Experiment 1. Food intake in mice that were fed a OO-based or BT-based HF diet (55% kcal as fat) supplemented with or without 1.0% GTE was measured on a per-cage basis throughout the study and represents cumulative energy intake.
